# Evaluation of depression, stress, and anxiety among women with subfertility during the COVID‐19 pandemic: A cross‐sectional study in Ahvaz, Iran

**DOI:** 10.1002/hsr2.967

**Published:** 2022-11-30

**Authors:** Poorandokht Afshari, Parvin Abedi, Reihaneh Sarizadeh, Mahmoud Maniati

**Affiliations:** ^1^ Midwifery Department, Reproductive Health Promotion Research Center Ahvaz Jundishapur University of Medical Sciences Ahvaz Iran; ^2^ Midwifery Department, Menopause Andropause Research Center Ahvaz Jundishapur University of Medical Sciences Ahvaz Iran; ^3^ Department of General Course, Reproductive Health Promotion Research Center Ahvaz Jundishapur University of Medical Sciences Ahvaz Iran

**Keywords:** anxiety, COVID‐19, depression, infertility, pandemic, stress

## Abstract

**Background and Aims:**

Some studies have shown that the levels of stress, anxiety, and depression have increased among subfertile women during the COVID‐19 pandemic. This study was designed to evaluate the levels of depression, anxiety, and stress among subfertile women during the COVID‐19 pandemic in southwest Iran.

**Method:**

This cross‐sectional study was conducted on 190 subfertile women from two infertility centers (Imam Khomeini and Jihad) in Ahvaz, Iran. A demographic questionnaire, and the depression, anxiety, stress scale (DASS‐21) were used to assess the level of depression, anxiety, and stress of subfertile women during the COVID‐19 pandemic. Data collection started in August 2021 and was completed in December 2021. Mean ± SD or *N* (%), and multiple linear regression were used to analyze the data.

**Result:**

Results showed that most women experienced moderate depression, anxiety, and stress. However, the percentage of women who experienced very severe anxiety was more than that for depression and stress, and 75 (39.5%) of women had all three disorders together. Anxiety was 0.176 units lower in women who were not affected by COVID‐19 (95% CI: −5.781 to −0.629). The depression was 0.216 units lower in women with good and moderate economic status (95% CI: −5.603 to −1.178).

**Conclusion:**

The results of this study showed that most studied women experienced moderate depression, anxiety, and stress, but the percentages of very severe anxiety were more than that for depression and stress. Poor economic status was also a strong predictor of depression among subfertile women. Infection with COVID‐19 increased the level of anxiety. Careful evaluation of subfertile women for mental health is recommended especially during crises such as the COVID‐19 pandemic.

## 1 INTRODUCTION

Infertility is defined as the couple's failure to achieve pregnancy after 12 months of having regular sexual intercourse and not using any contraception method.^1^ There are mainly two types of infertility, namely primary and secondary. In primary infertility, pregnancy has never been achieved, while in secondary infertility, there is at least one history of pregnancy.^1^ According to the World Health Organization, around 48 million couples and 186 million individuals in the world are living with infertility.^1^ The prevalence of infertility in Iran is reported to be 7.88%, of which 3.09% and 2.18% are of the primary and secondary types, respectively.^2^ A systematic review including 5874 Iranian participants showed that the prevalence of primary and secondary infertility was more in women than in men.^3^ While some couples with infertility can cope with this problem, the following have been reported to be among the negative consequences of this condition: reduced self‐esteem, feelings of failure, worry, fear, anxiety, loneliness, guilt or grief, sexual dysfunction, violence, depression, and anxiety.^4^, ^5^

In another systematic review, Kiani et al.^6^ found that the rate of depression in subfertile women varies from 21.01% to 52.21% according to the scale they used in their study. Also, the rate of depression among subfertile women living in low and middle‐income countries (44.32%) has been reported to be higher than that in high‐income countries (28.03%). Depression, anxiety, and stress may deteriorate the results of infertility treatment. There is evidence that couples with low levels of stress and anxiety have better assisted reproductive technology outcomes and higher pregnancy rates.^7^

Previous studies have found that subfertile women were more likely to have more anxiety and stress during the COVID‐19 pandemic. For example, Lablanche et al.^8^ found that during the COVID‐19 pandemic, subfertile women had more perceived stress compared to anxiety and depression (27 ± 6.75 vs. 7.58 ± 3.85 and 4.51 ± 3.48, respectively). Also, Cao et al.^9^ reported that the rate of anxiety among women who were in home quarantine was significantly more than that in subfertile women without home quarantine. Another study revealed that the infertility treatment of most subfertile women was stopped or postponed during COVID‐19 pandemic and this may be the cause for stress and anxiety, especially among older patients (older than 35 years old).^10^ According to a study conducted before pandemic in Iran, the rates of anxiety and depression among subfertile Iranian people were 49.6% and 33% respectively, and women were twice more likely have these disorders compared to men.^11^ Also, a systematic review and meta‐analysis including 31 studies showed that the prevalence of depression among subfertile women was 48.7%.^12^ Although, there are some evidence regarding stress, anxiety, and depression among subfertile women before the pandemic, there is paucity of information about the level of these mental disorders during COVID‐19 pandemic in Iran. Therefore, this study was designed to evaluate the levels of depression, anxiety, and stress among subfertile women during COVID‐19 pandemic in southwest of Iran.

## 2 RESEARCH DESIGN AND METHODS

This cross‐sectional study was conducted on 190 subfertile women who were recruited from two infertility centers (Imam Khomeini and Jihad Daneshgahi) in Ahvaz, Iran. Most of the subfertile women in this city are referred to these two clinics for infertility treatment. The design of this study was approved by the Ethics Committee of Ahvaz Jundishapur University of Medical Sciences (Ref. No: IR.AJUMS.REC.1400.333). All participants provided written informed consent before data collection.

### 2.1 Inclusion/exclusion criteria

Women who were under treatment in one of infertility centers and were willing to participate were recruited. Women who were under treatment for depression, anxiety, or stress, addicted women, and those who had gone through a critical event during past 6 months were excluded from the study.

### 2.2 Sample size

According to previous study^13^ that showing the 46% prevalence of anxiety in subfertile women, and using the following formula:

$$n=\frac{z_{1-\alpha /2}^{2}\times p(1-p)}{d^{2}}$$

Z=1.96 d=0.05 p=0.46

The sample size of this study was calculated to be 195.

### 2.3 Setting

Two infertility management and treatment centers (Imam Khomeini and Jihad Daneshgahi) that are referral centers for subfertile couples in Ahvaz, Iran were chosen for sampling. All eligible women who provided consent were requested to complete a demographic questionnaire and the depression, anxiety, stress scale (DASS‐21). One of the researchers (R. S.) was available in case women had any question. Data collection was started in August 2021 and completed in December 2021.

### 2.4 Instruments

A demographic questionnaire was used to collect demographic data as well as data about the participants' infertility and COVID‐19 infection. This questionnaire included questions about age, age of the husband, educational attainment, occupation, duration of fertility, economic status, education, and occupation of the husband. The second section of this questionnaire was about COVID‐19 infection in the participating women and their husbands, hospitalization, and vaccination. The questionnaire was given to five faculty members and their corrective comments were applied to it (content validity).

The DASS‐21 was used to assess the level of depression, anxiety, and stress of the participants during the COVID‐19 pandemic. This scale has 21 questions, dedicating seven questions to each area of depression, anxiety, and stress. The range of scores varies from zero for “never” to 3 for “almost always.” According to answers, the scores between 0 and 4, 5 and 6, 7 and 10, and 11 and 13 were considered to represent normal, mild, moderate, and severe depression respectively. Scores between 0 and 3, 4 and 5, 6 and 7, and 8 and 9 indicated normal, mild, moderate, and severe anxiety, respectively.^14^ The psychometrics assessment of the Persian version of this questionnaire was done by Sahebi et al.^15^

#### 2.4.1 Variables

COVID‐19 home quarantine was considered as independent variable and stress, anxiety, and depression in subfertile women were considered as dependent variables.

### 2.5 Statistical analysis

All data were entered into SPSS version 23 (IBM Corp.; IBM SPSS Statistics for Windows). The mean ± SD or *N* (%) was used for presenting continuous or categorical data. The normality of data was tested and first we used univariate linear regression to check the relationship of demographic variables with depression, anxiety, and stress. Those variables that remained significant in the univariate model were entered to multiple linear regression.

Multiple linear regression was used to test the association of depression, anxiety, and stress with some demographic factors. *p* < 0.05 was considered statistically significant.

## 3 RESULTS

### 3.1 Demographic characteristics

Overall, 195 women were recruited in this study, of whom 5 did not complete the questionnaires, and we analyzed 190 questionnaires. Demographic characteristics of the participants are presented in Table 1. The participants' mean age was 31.46 ± 6.01 years, and their infertility duration was 4.92 ± 3.84 years. Most of the participants (60%) had a university degree, and more than half of the participants 104 (54.7%) were housewives.

**Table 1:** Demographic characteristics of participants (*n* = 190)

Variables	Mean ± SD
Age (year)	31.46 ± 6.01
Age of husband (year)	35.49 ± 5.90
Length of marriage (year)	7.93 ± 5.21
The duration of failed pregnancy attempts (year)	4.92 ± 3.84
Duration of using contraceptive methods (year)	3.02 ± 2.44
Duration of infertility treatment (year)	3.93 ± 3.52
	*N* (%)
Education	
High school	24 (12.6)
Diploma	52 (27.4)
University degree	114 (60)
Husband's education	
High school	29 (15.3)
Diploma	61 (32.1)
University degree	100 (52.6)
Occupation	
Housewife	104 (54.7)
Employee	86 (45.3)
Occupation of husband	
Self‐employed	94 (49.5)
Employed	80 (42.1)
Unemployed	16 (8.4)
Economic status	
Poor	24 (12.6)
Moderate	127 (66.8)
Well‐off	39 (20.5)

### 3.2 Infertility related factors and COVID‐19 infection

Table 2 shows the infertility related factors and history of COVID‐19 infection among the participants. As evident from this table, most women did not report a previous pregnancy, the cause of their infertility was mostly female factor (32.6%), and 122 (64.2%) had a history of infertility treatment. Also, 87 (45.8%) of the participants had been infected with COVID‐19 infection, but only 18 (9.5%) of them were hospitalized, and 121(63.7%) received COVID‐19 vaccines.

**Table 2:** Infertility related factors and COVID‐19 infection in subfertile women (*n* = 190)

Variables	*N* (%)
Did you have any pregnancy?	
No	101 (53.2)
Yes	89 (46.8)
The cause of infertility	
Female factor	62 (32.6)
Male factor	49 (25.8)
Couple factor	25 (13.2)
Unknown	54 (28.4)
Infertility treatment before pandemic	
No	68 (35.8)
Yes	122 (64.2)
Delay in infertility treatment due to pandemic	
No	85 (44.7)
Yes	105 (55.3)
Were you infected with COVID‐19 infection?	
No	103 (54.2)
Yes	87 (45.8)
Was your husband infected with COVID‐19 disease?	
No	108 (56.8)
Yes	82 (43.2)
Were you hospitalized due to COVID‐19 infection?	
No	172 (90.5)
Yes	18 (9.5)
Did you receive any COVID‐19 vaccines?	
No	69 (36.3)
Yes	121 (63.7)
Did you receive any consultation?	
No	107 (56.3)
Yes	83 (43.7)

### 3.3 Stress, anxiety, and depression among subfertile women

Table 3 shows the responses of women to DASS‐21 questionnaire during pandemic and comparison of them with before pandemic. As evident from this table, in response to questions about depression, most women had symptoms of depression “almost always” or “often.” In response to anxiety questions, 56.8% of women almost always were worried about situations in which they might panic and make a fool of myself. In response to questions about stress, 53%, 56%, 56%, and 58% of participants almost always experienced the following feelings: “I felt I was not worth much as a person,” “I was aware of the action of my heart in the absence of physical exertion,” “I felt scared without any good reason,” and “I felt that life was meaningless” respectively.

**Table 3:** Prevalence of depression, anxiety, and stress during COVID‐19 pandemic according to DASS‐21 questionnaire

	Almost always	Often	Sometimes	Never
Questions	*N* (%)			
1 I found it hard to wind down	56 (29.5)	74 (38.9)	42 (22.1)	18 (9.5)
2 I was aware of dryness of my mouth	89 (46.8)	65 (34.2)	26 (13.7)	10 (5.3)
3 I could not seem to experience any positive feeling at all	74 (38.9)	60 (31.6)	36 (18.9)	20 (10.5)
4 I experienced breathing difficulty	118 (62.1)	39 (20.5)	21 (11.1)	12 (6.3)
5 I found it difficult to work up the initiative to do thing	67 (35.3)	68 (35.8)	41 (21.6)	14 (7.4)
6 I tended to over‐react to situations	63 (33.2)	70 (36.8)	30 (15.8)	27 (14.2)
7 I experienced trembling	106 (55.8)	40 (21.1)	21 (11.1)	23 (12.1)
8 I felt that I was using a lot of nervous energy	76 (40)	44 (23.2)	34 (17.9)	36 (18.9)
9 I was worried about situations in which I might panic and make a fool of myself	108 (56.8)	43 (22.6)	18 (9.5)	21 (11.1)
10 I felt that I had nothing to look forward to	94 (49.5)	49 (25.8)	23 (12.1)	24 (12.6)
11 I found myself getting agitated	74 (38.9)	64 (33.7)	39 (20.5)	13 (6.8)
12 I found it difficult to relax	77 (40.5)	56 (29.5)	39 (20.5)	18 (9.5)
13 I felt down‐hearted and blue	79 (41.6)	50 (26.3)	37 (19.5)	24 (12.6)
14 I was intolerance of anything that kept me from getting on with what I was doing	46 (24.2)	70 (36.8)	46 (24.2)	28 (14.7)
15 I felt I was close to panic	79 (41.6)	50 (26.3)	34 (17.9)	27 (14.2)
16 I was unable to become enthusiastic about anything	81 (42.6)	57 (30)	37 (19.5)	24 (12.6)
17 I felt I was not worth much as a person	101 (53.2)	43 (22.6)	29 (15.3)	17 (8.9)
18 I felt that I was rather touchy	49 (25.8)	60 (31.6)	38 (20)	43 (22.6)
19 I was aware of the action of my heart in the absence of physical exertion	107 (56.3)	36 (18.9)	25 (13.2)	22 (11.6)
20 I felt scared without any good reason	107 (56.3)	47 (24.7)	20 (10.5)	16 (8.4)
21 I felt that life was meaningless	111 (58.4)	43 (22.6)	20 (10.5)	16 (8.4)

Abbreviation: DASS‐21, depression, anxiety, stress scale.

**Figure 1:**
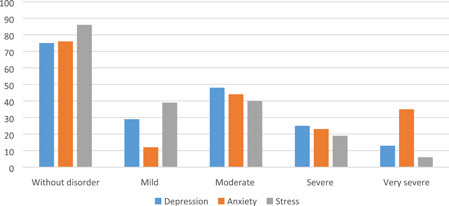
Frequency of depression, anxiety, and stress during COVID‐19 pandemic

Figure 1 shows the frequency of depression, anxiety, and stress during pandemic. As this figure shows, most women experienced moderate depression, anxiety, and stress. However, the percentage of women who experienced very severe anxiety was more than that for depression and stress. Regarding the simultaneous occurrence of all three mental disorders, 41 (21.6) of women had two symptoms of depression and anxiety at the same time, and 75 (39.5) had all three disorders together (data are not shown in tables).

### 3.4 Association of some demographic variables with depression, anxiety, and stress in subfertile women

Table 4 shows the association of some demographic variables with depression, anxiety, and stress in subfertile women during COVID‐19 pandemic using multiple linear regression. As this table shows, anxiety was 0.176 units lower in women who were not affected with COVID‐19 (95% CI: −5.781 to −0.629). The depression was 0.216 units lower in women with good and moderate economic status (95% CI: −5.603 to −1.178).

**Table 4:** The relationship between some demographic variables and depression, anxiety, and stress in subfertile women during the COVID‐19 pandemic (multiple linear regression)

	Unstandardized coef		Standardized coef			95% confidence interval	
Variable	*β*	SE	*β*	*t*	Sig	Lower	Upper
Affected with COVID‐19 and anxiety	−3.205	1.306	−0.176	−2.454	0.015	−5.781	−0.629
Economic status and depression	−3.391	1.122	−0.216	−3.023	0.003	−5.603	−1.178

Abbreviations: Coef, coefficient; SE, standard error.

## 4 DISCUSSION

This study was designed to evaluate the levels of depression, anxiety, and stress of subfertile women during the COVID‐19 pandemic in Ahvaz, Iran. In line with other studies, our results showed that the cause of infertility was mostly a female factor, and most of the participants had a history of infertility treatment.^16^

The results of the present study showed that around half of the women studied did not have depression and anxiety, but the other half reported moderate and severe depression and anxiety. Also, half of the participants were equally affected by mild and moderate stress.

In a systematic review by Kiani et al.^6^ the prevalence of depression among subfertile women was 44.32% in low‐ and middle‐income countries and 28.03% in developed countries, which is similar to what we found in our study. Also, Rasekh Jahromi et al.^17^ showed that subfertile women whose infertility treatment was postponed because of the COVID‐19 pandemic, perceived more depression compared with those who did not start their infertility treatment. Studies before the pandemic showed high level of depression among subfertile Iranian women,^18^ which mostly were mild depression. But in the present study half of the participants had moderate or severe depression.

Lakatos et al.^19^ found that subfertile women experienced more depression and anxiety than fertile women did. Depression was significantly related to age, social concern, sexual function, and financial stress. It is not clear if the depression is the cause of infertility or whether infertility causes depression and anxiety. However, it is clear that subfertile women have more depression and anxiety, and this may negatively affect their conception.^20^ Cao et al.^9^ reported that women who were in the home quarantine had more anxiety, and women with a longer infertility duration were more likely to have anxiety.

In contrast with our findings, Galhardo et al.^21^ found that the levels of depression and anxiety among subfertile women during the COVID‐19 pandemic remained unchanged, and they even reported a reduced level of stress in this population. The reason for this discrepancy may be due to the fact that the Iranian government started its vaccination program almost 1 year after the beginning of the pandemic, and this may have caused emotional distress.

In line with other studies,^22^ the results of the present study showed that failure to conception increased the level of depression. Also, women with moderate or good economic status experienced less depression compared to those with poor economic status. In a study on 385 subfertile women in Gaza Strip, Elsous et al.^23^ found that around half of the women studied had depression, and that duration of marriage, and primary infertility were the predictors of depression. Also, Esposito et al.^24^ in a study on 627 subfertile couples found that home quarantine increased the level of anxiety, stress or depression, and that more women were affected compared with men. Our findings are in line with those of Esposito et al. except that we did not assess the level of anxiety, stress, or depression among male partners.

Unlike our study, other studies did not find any relationship between economic status and depression, anxiety, or stress, which could be attributed to the fact that in Iran, the expenses of infertility are not covered by insurance, and that some people lost their jobs and had reduced income during the COVID‐19 pandemic, and this may have caused depression.

### 4.1 Limitations of the study

In this study we did not recruit women randomly and this may affect the generalizability of the findings. Furthermore, we did not assess the level of depression, stress, and anxiety of male partners.

## 5 CONCLUSION

The results of this study showed that most subfertile women experienced moderate depression, anxiety, and stress during the pandemic. However, the percentage of women who experienced very severe anxiety was more than that for depression and stress. Poor economic status was a strong predictor for depression in subfertile women. Finally, women infected with COVID‐19 infection had more anxiety. Health policymakers should devise careful strategies to improve mental health of subfertile women, especially during crises such as COVID‐19 pandemic.

## AUTHOR CONTRIBUTIONS

**Poorandokht Afshari**: conceptualization; formal analysis; funding acquisition; methodology; project administration; supervision; writing – review & editing. **Parvin Abedi**: conceptualization; formal analysis; investigation; methodology; software; supervision; writing – original draft; writing – review & editing. **Reihaneh Sarizadeh**: conceptualization; data curation; formal analysis; methodology; software; validation; writing – review & editing. **Mahmoud Maniati**: conceptualization; formal analysis; methodology; software; validation; writing – original draft; writing – review & editing.

## CONFLICT OF INTEREST

The authors declare no conflict of interest.

## TRANSPARENCY STATEMENT

The corresponding author Parvin Abedi affirms that this manuscript is an honest, accurate, and transparent account of the study being reported; that no important aspects of the study have been omitted; and that any discrepancies from the study as planned (and, if relevant, registered) have been explained.

## Supporting information

Supporting information.

## Data Availability

Data used in this study will be available upon reasonable request from corresponding author.
